# Investigating mediating influences of β‐amyloid on the relationship between marital dissolution and cognition

**DOI:** 10.1002/alz.092267

**Published:** 2025-01-03

**Authors:** Avinash Chandra, Rifah Anjum, Sheena Waters, Petroula Proitsi, Laura J Smith, Charles R Marshall

**Affiliations:** ^1^ Queen Mary University of London, London United Kingdom; ^2^ Centre for Preventive Neurology, Wolfson Institute of Population Health, Queen Mary University of London, London United Kingdom; ^3^ Queen Mary University of London, London, EC1M 6BQ United Kingdom

## Abstract

**Background:**

Research has demonstrated that spousal loss through widowhood or divorce are associated with an increased risk of dementia and deteriorated cognitive performance. This is likely due to high levels of stress characteristic of these life events. Evidence suggests that neuropathology typically seen in Alzheimer’s disease, for example, β‐amyloid (Aβ), may be a by‐product of chronic stress. However, whether Aβ facilitates links between disruptive marital transitions and poor cognition remains unknown. The aim of the current study was to explore whether Aβ pathology could mediate associations between marital dissolution, either through divorce or widowhood, and domain‐specific cognitive performance in a cohort of cognitively normal (CN) subjects.

**Method:**

Data was included for 543 participants in the Alzheimer’s Disease Neuroimaging Initiative classified as CN that had data on self‐reported marital status (married, divorced or widowed) and Aβ PET imaging. Aβ levels were quantified by Centiloid values. Cognitive performance data on executive functioning and episodic memory was available for all participants. Multiple linear regression was utilised for exploring inter‐relationships between marital dissolution, Aβ burden, and cognition, whilst follow‐up mediation analysis examined indirect effects of Aβ on the association between marital status and cognitive performance.

**Results:**

Associations were found between elevated Aβ levels and both marital dissolution and poorer memory. Results from causal mediation analysis evidenced that Aβ was a significant mediator for the relationship between marital dissolution and episodic memory. These results were confirmed using the Baron and Kenney criteria for a partial mediation effect.

**Conclusion:**

This study provides novel evidence that the accumulation of Aβ pathology in the brain may be a pathway through which the stress of marital dissolution acts on memory abilities. This may have further implications for dementia risk, but future research is needed. Findings highlight that interventions after spousal loss may be helpful for the preservation of brain health.

